# Water evaporation from solute-containing aerosol droplets: Effects of internal concentration and diffusivity profiles and onset of crust formation

**DOI:** 10.1063/5.0060080

**Published:** 2021-09-14

**Authors:** Majid Rezaei, Roland R. Netz

**Affiliations:** Fachbereich Physik, Freie Universität Berlin, 14195 Berlin, Germany

## Abstract

The evaporation of droplets is an important process not only in industrial and scientific applications, but also in the airborne transmission of viruses and other infectious agents. We derive analytical and semi-analytical solutions of the coupled heat and mass diffusion equations within a spherical droplet and in the ambient vapor phase that describe the evaporation process of aqueous free droplets containing nonvolatile solutes. Our results demonstrate that the solute-induced water vapor-pressure reduction considerably slows down the evaporation process and dominates the solute-concentration dependence of the droplet evaporation time. The evaporation-induced enhanced solute concentration near the droplet surface, which is accounted for using a two-stage evaporation description, is found to further slow-down the drying process. On the other hand, the presence of solutes is found to produce a lower limit for the droplet size that can be reached by evaporation and, also, to reduce evaporation cooling of the droplet, which tend to decrease the evaporation time. Overall, the first two effects are dominant, meaning that the droplet evaporation time increases in the presence of solutes. Local variation of the water diffusivity inside the droplet near its surface, which is a consequence of the solute-concentration dependence of the diffusion coefficient, does not significantly change the evaporation time. Crust formation on the droplet surface increases the final equilibrium size of the droplet by producing a hollow spherical particle, the outer radius of which is determined as well.

NOMENCLATURE

**Symbols and notations**

$c_{g}$

: Saturated water vapor concentration

$c_{s}$

: Solute concentration

$c_{v}$

: Water vapor concentration

$c_{w}$

: Liquid water concentration

$c_{0}$

: Ambient water vapor concentration

$c_{s}^{ev}$

: Solute concentration at the equilibrium state

$c_{s}^{i}$

: Initial solute concentration

$c_{s}^{\text{max}}$

: Solute concentration at the onset of crust formation

$c_{w}^{i}$

: Initial water concentration inside the droplet

$D_{w}$

: Water diffusion constant in air

$D_{s}^{l}$

: Solute diffusion constant in liquid water

$D_{w}^{l}$

: Water diffusion constant in pure liquid water

$D_{w}^{\mathrm{sol}}$

: Water diffusion constant in water solution

$D_{w}^{\mathrm{sol},i}$

: Initial water diffusion constant inside the droplet

$h_{ev}$

: Molecular evaporation enthalpy of water

J

: Total evaporation flux

j

: Evaporation flux density

$J_{h}$

: Total heat flux into the droplet

$k_{c}$

: Condensation reaction rate constant

$k_{e}$

: Evaporation reaction rate constant

$N_{A}$

: Avogadro constant

R

: Droplet radius

r

: Radial distance from the droplet center

$R_{ev}$

: Equilibrium radius of the droplet

$R_{i}$

: Radius of the inner core

$R_{0}$

: Initial radius of the droplet

$R^{{}^{\circ}}$

: Droplet radius at the end of the first drying stage

$R_{i}^{{}^{\circ}}$

: Radius of the inner core at the end of the first drying stage

RH

: Relative humidity

t

: Time

$T_{s}$

: Droplet surface temperature

$T_{0}$

: Ambient temperature

$v_{w}$

: Liquid water molecular volume

γ

: Activity coefficient of water

ΔT

: Evaporation induced temperature reduction at the droplet surface

$\lambda_{\mathrm{air}}$

: Heat conductivity of air

$\tau_{ev}$

: Evaporation time of a pure water droplet

${\bar{\tau }}_{ev,s}$

: Evaporation time of a solute-containing droplet in the presence of internal concentration gradients

$\tau_{ev,s}^{1st}$

: The time of the first drying stage

$\tau_{ev,s}^{2nd}$

: The time of the second drying stage

Φ

: Momentary volume fraction of solutes

$\Phi_{ev}$

: Final equilibrium volume fraction of solutes

$\Phi_{i}$

: Volume fraction of solutes in the inner core

$\Phi_{0}$

: Initial volume fraction of solutes

$\Phi_{0}^{\tau_{\text{max}}}$

: Initial solute volume fraction at which the evaporation time is maximal

$\psi_{c}$

: Evaporation-cooling factor

$\psi_{c}^{\Phi_{0}\to 0}$

: Evaporation-cooling factor in the absence of solutes

**Numerical constants**

$c_{g}$

: $7.69\times 10^{23}\;m^{-3}$ at 25 °C and $1.62\times 10^{23}\;m^{-3}$ at 0 °C (Ref. 5)

$D_{w}$

: $2.5\times 10^{-5}\;m^{2}/s$ at 25 °C (Ref. 1)

$D_{w}^{l}$

: $2.3\times 10^{-9}\;m^{2}/s$ at 25 °C (Ref. 2)

$h_{ev}$

: $7.3\times 10^{-20}\;J$ at 25 °C (Ref. 5)

$k_{c}$

: 370 m/s at 25 °C (Ref. 6)

$v_{w}$

: $3\times 10^{-29}\;m^{3}$ at 25 °C (Ref. 3)

$\lambda_{\mathrm{air}}$

: 0.026 W/m K at 25 °C (Ref. 4)

## I. INTRODUCTION

Evaporation from solute-containing droplets is an important process in many industrial and scientific applications ranging from pharmacology, agriculture, food, and cosmetics production to medical, biochemical, material, and soil sciences.^7–12^ Depending on the rate of the solvent evaporation from the droplet surface and the diffusive transport rate of solutes inside the droplet, the drying process can lead to particles with different morphologies and thus different properties and functionalities. It is, therefore, of importance to investigate the morphological evolution during evaporation, which is affected by solute diffusion inside the droplet. These processes are controlled by various parameters, such as temperature and relative humidity of the ambient air, the physical–chemical properties of solvent and solutes, the initial volume fraction of solutes, and the initial size distribution of droplets. Theoretical investigations are, therefore, required to explain how the droplet evaporation kinetics depends on all the relevant parameters.

In addition, the droplet-evaporation process plays an important role in the transmission of infectious diseases and respiratory viruses via the airborne route,^6,13–16^ which is the main motivation of the present study. It is known that saliva is primarily composed of water, but additionally includes a variety of organic and inorganic substances such as salt, proteins, peptides, mucins, virions, etc.^17^ The presence of such nonvolatile components produces a lower limit for the water content of a saliva droplet that can be reached by evaporation. Accordingly, water evaporation from a saliva droplet might eventually produce a droplet nucleus, which is a small light particle with a minimal moisture level that stays floating in air for a long time.^18^ The creation of such droplet nuclei, which is more likely in the case of small droplets due to their sufficiently long sedimentation time,^19–21^ can significantly influence the infection risk from virus-containing respiratory droplets, especially in indoor environments,^14,18^ by increasing the sedimentation time of droplets.^6^ It is, therefore, of utmost importance to investigate how respiratory droplets dry out and how their evaporation kinetics is affected by different factors such as the droplet composition and its initial size, the ambient relative humidity, temperature, and the surrounding air flow.

Although there are many studies^11,22–25^ concerning the drying process of droplets placed on solid surfaces, also known as sessile droplets, fewer investigations have been conducted on free droplets surrounded by air. Indeed, designing and conducting experiments on free droplets faces many challenges, mostly since such droplets are ephemeral and difficult to follow.^8^ So far, a few experiments have been conducted and are used alone or in combination with theoretical analysis to measure time-dependent properties of evaporating or condensing free droplets, such as their size,^26,27^ viscosity,^26,28^ and hygroscopicity,^29–31^ as well as the solvent diffusion coefficient inside droplets.^32,33^ Also, the morphology of aerosols has been investigated using different experimental methods.^27,34^ Various physical–chemical effects and mechanisms that affect the droplet evaporation process must be accounted for in the interpretation of these experiments. Therefore, analytical and numerical modeling methods must be used to better understand the details of the evaporation mechanism that is relevant for sedimenting droplets. Although modeling of free droplets does not face complexities due to substrate–droplet interactions that control the droplet shape at the solid/liquid/air interface^35,36^ and Marangoni flow,^37,38^ which occur in the case of sessile droplets, other factors such as evaporation-induced concentration gradients inside the droplet^39^ and the possibility of crust formation on the droplet surface,^40^ which are consequences of the increasing solute surface concentration during evaporation,^41^ cause difficulties even in the modeling of free droplets in the presence of solutes. In addition, physical and chemical properties of the drying droplets, such as the internal viscosity,^42,43^ the diffusivity of solvent and solutes in the liquid phase,^43^ and the activity coefficient of the solvent,^44^ are dependent on the local concentration of solutes (and, consequently, on both position and time), which makes the problem rather complex. A challenge in modeling evaporating droplets is, therefore, to account for the concentration gradients created and developed within droplets during the solvent evaporation process. Such concentration gradients are not only relevant for solutions containing slowly diffusing species, e.g., polymers and proteins, but also for relatively quickly diffusing solutes such as NaCl.^8^ In fact, internal concentration gradients have been experimentally observed for drying respiratory fluids suspended on superhydrophobic substrates using optical and fluorescence microscopy.^45^

There are a few studies that address concentration gradients inside a solute-containing drying droplet^39,46–48^ and propose analytical and numerical methods for modeling the evaporation process before^47–50^ and after^40,49,51^ crust formation. Handscomb *et al.*^52^ proposed a numerical model consisting of a set of advection–diffusion equations coupled to ordinary differential equations describing the particle size and temperature to simulate the drying of droplets that contain solid particles prior to crust formation. Solute nucleation and crust formation in drying free droplets were numerically modeled by Robinson *et al.*^53^ using a single-particle approach based on the diffusion equation. Diffusion through atmospheric aerosols has been numerically simulated using core–shell multilayer models such as the KM-GAP model proposed by Shiraiwa *et al.*^54^ based on the PRA (Poschl-Rudich-Ammann) framework,^55^ the model proposed by Fowler *et al.*^56^ based on the Maxwell–Stefan diffusion framework, and the Fi-PaD model proposed by O'Meara *et al.*^57^ based on the Fickian diffusion framework. The latter two models can be used for droplets containing both non- and semi-volatile solutes. For example, Ingram *et al.*^26^ simulated evaporation kinetics of droplets that contain water and semi-volatile organic compounds using the Fi-PaD model. Also, a few analytical or semi-analytical approaches have been proposed to treat solute diffusion inside a drying droplet, most of which use a fixed droplet shrinking rate. In a series of works by Sazhin *et al.*^58^ and Gusev *et al.*,^59^ it was assumed that the droplet radius decreases linearly in time. This assumption, however, holds only for very small droplets in the so-called reaction-rate-limited regime.^6^ In fact, for industrial droplet-drying processes and airborne transmission of infections, the initial radius of the drying droplets is typically larger than the threshold radius below which the reaction-rate-limited scenario is valid [∼68 nm for pure water droplets at 25 °C (Ref. 6)] In other droplet evaporation models,^46,60–64^ the analytical calculations are based on the so-called *R*^2^-law, which assumes that the droplet temperature and thereby also the droplet evaporation rate remain constant during the evaporation process and, thus, the droplet surface decreases linearly in time.^65^ Although this approximation describes the evaporation-induced shrinkage of pure solvent droplets accurately, it cannot predict the osmotic slowing down of the evaporation process due to the solute-induced water vapor-pressure reduction.^6^ We show in this paper that the osmotic effect plays a key role in determining the droplet evaporation time. Deviations from the *R*^2^-law were accounted for in a model developed by Eslamian *et al.*,^66,67^ where the droplet evaporation rate was calculated from a combination of Fick's law^68^ and kinetic theory.^69^ The equations provided by this model are, however, not analytically solvable. Our model describes the evaporation process of solute-containing droplets and, in particular, accounts for the solute-induced deviation from the classical *R*^2^-law model.

**FIG. 1.**
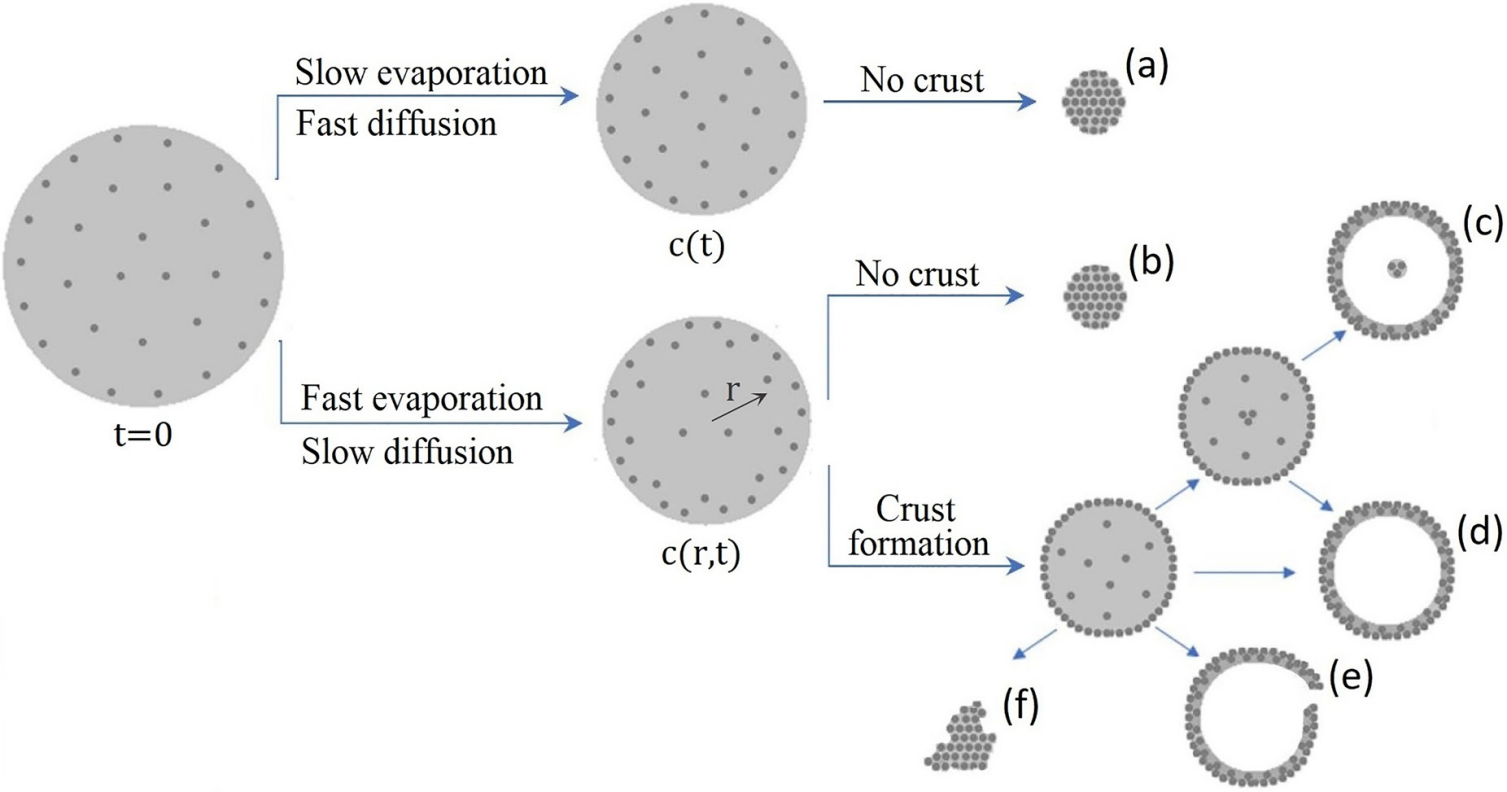
Schematic illustration of possible scenarios for the morphological evolution of a solute-containing droplet during the drying process. When the solvent evaporation is very slow (or the internal particle diffusion inside the droplet is sufficiently rapid), the solute particles have enough time to redistribute in the liquid phase and thus the internal solute concentration remains uniform throughout the evaporation process (except the possibility of two-phase coexistence when the critical supersaturation concentration is reached). In such case, a solid particle will be produced at the end of the drying process [scenario (a)]. In the case of fast solvent evaporation, however, the shrinkage rate of the droplet radius overcomes the diffusion rate of the solutes in the solution, which leads to an increased solute concentration at the droplet surface. In this case, depending on the type of the solutes and their solubility limit in the solvent, the dry particle produced at the end of the evaporation process might be a solid particle [scenario (b)] or a hollow particle that may [scenario (c)] or may not [scenario (d)] contain small agglomerates of solutes.^40^ Alternatively, a collapsed dry particle [scenario (e)] will be produced when the crust formed at the droplet surface cannot withstand the pressure difference caused by the continued internal solvent evaporation.^70^ Very fast solvent evaporation can lead to non-uniform drying conditions due to concentration inhomogeneities in the droplet.^70^ In such case, an irregularly shaped dry particle, also known as a wrinkled particle, will be formed at the end of the drying process [scenario (f)].

The evaporation-induced concentration gradients inside drying droplets are found to strongly affect the drying process not only by decreasing the evaporation rate,^48^ but also by influencing the morphological evolution of the drying droplet^40,70^ and, in particular, the shape and properties of the particles produced at the end of the drying process. An important physical parameter is the ratio of the evaporation rate to the diffusive transport rate of solute particles inside the droplet,^71^ which is known as the Péclet number.^64,72^ When the evaporation process is very slow or, alternatively, the internal diffusion is very fast, the solute particles have enough time to redistribute during the drying process. In this case, the solute particles remain evenly distributed within the droplet to form a full solid particle at the end of the drying process [Fig. 1, scenario (a)]. In the case of fast evaporation, however, the solvent evaporation from the droplet surface increases the solute concentration at the surface and creates a concentration gradient in the droplet [see Fig. 1, scenarios (b)–(f)]. If the critical supersaturation concentration at which phase separation occurs is not reached during evaporation, the concentration gradient gradually disappears as the evaporation proceeds and a full solid particle remains at the end of the drying process [Fig.1, scenario (b)]. When the critical supersaturation concentration is reached during evaporation, however, two-phase coexistence is obtained due to radial de-mixing^34^ and the solute particles at the droplet surface form a solid crust [see Fig. 1, scenarios (c)–(e)], which is expected to mainly affect the drying mechanism and to considerably slow down the evaporation process.^70^ The fate of the crust and the physical and morphological properties of the dried particle formed at the end of the evaporation process are affected by various parameters such as droplet diameter, initial solute concentration, solvent composition, and drying rate.^27^

Depending on the type of solutes, the crust that forms might be either a dry crust (expected for salty droplets)^8,73^ or a gel-like wet skin consisting of polymers or proteins and other suspended particles.^74,75^ In the presence of a dry crust, the liquid from the internal liquid core reaches the crust surface through capillary action within the crust pores.^70,76^ As the evaporation proceeds, the crust will become thicker and the crust pores will lengthen, which increases the resistance to heat and mass transfer and decreases the evaporation rate. In the case of a wet crust, water evaporation continues via diffusion through the gel crust.^74^ Since the diffusion coefficient and the concentration gradient within the gel phase are both much lower than in solution, the evaporation rate is expected to fall after the formation of a wet crust as well. Regardless of the crust type, crust formation is expected to determine the final morphology of the droplet by producing a hollow structure, with a size larger than expected in the absence of a crust. Depending on the type of the solute particles within the droplet, the hollow structure produced at the end of the evaporation process may or may not contain small agglomerates of solutes^40^ [Fig. 1, scenarios (c) and (d), respectively]. Also, a crust collapse might occur when the crust cannot withstand the pressure difference caused by the continued solvent evaporation^70^ [Fig. 1, scenario (e)]. Another scenario accounts for non-uniform drying conditions due to amplification of concentration inhomogeneities over the droplet surface, which is probable when the solvent evaporation is very fast. In such case, an irregularly shaped dry particle, also known as a wrinkled particle, will form at the end of the drying process^70^ [Fig. 1, scenario (f)].

In this paper, a droplet evaporation model is presented by solving the heat and mass diffusion equations analytically or semi-analytically to describe the various effects nonvolatile solutes have on the drying process. Starting from the simple model scenario that only accounts for the solute-induced minimal water concentration inside the droplet, we gradually add complexities to our model to address different factors associated with the presence of solutes, i.e., the solute-induced water vapor-pressure reduction, effects of solutes on evaporation cooling, evaporation-induced water concentration gradients inside the droplet, and the dependence of the internal water diffusivity on the solute concentration. We also include the possibility of crust formation in our calculations to evaluate its effect on the droplet equilibrium size. All our calculations are done in the one-phase regime, meaning from the beginning of the evaporation process up to the point where crust formation sets in at the surface, and for a single droplet at rest, which defines the so-called stagnant-flow approximation and is valid for droplet radii up to 60 *μ*m.^6^ The time-dependent solute and solvent densities of the droplet are described in terms of the time-dependent solute volume fraction of the droplet, assuming ideal mixing of solute and solvent. Our results indicate that the presence of solutes causes a reduction of the evaporation rate and thereby also of the evaporation-cooling of the droplet not only by reducing the water vapor pressure, but also by inducing a locally reduced water concentration inside the droplet near the surface, which arises from the finite diffusivity of water and solutes in the droplet. Any factor that reduces the internal water and solute diffusivity, such as the presence of strongly hydrated solutes inside the droplet, intensifies this effect and thus further slows down the evaporation process. The time-dependent local variation of the internal water diffusivity, which occurs especially near the droplet surface due to the increased solute concentration, is, however, found to play only a minor role in determining the droplet evaporation time. Crust formation is suggested to affect the morphology of the final droplet nuclei by producing a hollow particle with a size larger than expected in the absence of a crust. The size of the hollow particle formed after crust formation follows approximately from our analytical model.

## II. RESULTS AND DISCUSSION

The main objective of the present study is to model water evaporation from a solute-containing aqueous droplet that is sedimenting in air. For this, the time-dependent water–vapor concentration profile outside the droplet and the liquid–water and solute concentration profiles inside the droplet are determined from the diffusion equation, which for spherical symmetry depends on the radial coordinate and reads

$$\frac{dc_{i}(r,t)}{dt}=\frac{1}{r^{2}}\frac{\partial }{\partial r}\left(D_{i}[c_{i}\left(r,t\right)]r^{2}\frac{\partial c_{i}(r,t)}{\partial r}\right),$$ (1)

where the subscript i denotes different molecular species (i.e., water vapor outside the droplet, liquid water, and solutes inside the droplet), $c_{i}\left(r,t\right)$ is the concentration profile, and $D_{i}[c_{i}\left(r,t\right)]$ is the respective molecular particle diffusion coefficient that in general depends on the concentration and, consequently, on time and position. As discussed in supplementary material Sec. B.1, the coupled diffusion equations for solvent and solutes within an incompressible solution are simultaneously solvable only when the diffusivities of the two components are the same. In the presence of slowly diffusing solutes, such as polymers or proteins, the water dynamics becomes slaved to the solute dynamics and thus one can approximately describe the diffusion dynamics by a single diffusion equation with a reduced effective diffusion coefficient, which is the approach we are taking. The exact problem is much more complicated and requires the solution of two coupled diffusion equations while accounting for the finite solution compressibility.

An important factor that affects the droplet evaporation rate is the evaporation-induced cooling of the droplet, which decreases the temperature and thereby also the water vapor concentration at the droplet surface. To account for this, one needs to calculate the temperature distribution around the evaporating droplet from the heat-conduction equation in radial coordinates,

$$\frac{\lambda }{r^{2}}\frac{d}{dr}r^{2}\frac{d}{dr}T\left(r,t\right)=C_{P}\frac{d}{dt}T\left(r,t\right),$$ (2)

with λ and $C_{P}$ being the heat conductivity and the heat capacity of air, respectively. In the present study, we ignore temperature gradients inside the droplet and thus the heat-conduction equation is solved only outside the droplet.

In the following, the results obtained from solutions of the above equations at various approximation levels are discussed. Derivations of all equations are provided in the supplementary material.

### A. Droplet evaporation in the presence of nonvolatile solutes assuming that the internal water diffusion is sufficiently rapid

For later comparison with our main results, we first assume that the particle diffusion inside the droplet is sufficiently rapid, so that the water concentration at the droplet surface equals the mean water concentration in the droplet. If one furthermore assumes that the water diffusion in the vapor phase is fast so that at each instant of time a stationary water–vapor concentration profile is obtained, the time-dependent variation of the droplet radius due to the water evaporation results from the stationary solution of the coupled water–vapor-diffusion and heat-conduction equations [Eqs. (1) and (2)] outside the droplet, considering total water mass conservation. As detailed in supplementary material Sec. A, the droplet radius *R* is determined by the differential equation

$$\frac{dR(t)}{dt}=-\psi_{c}\left[\Phi \left(R(t)\right)\right]\left(\frac{\gamma D_{w}c_{g}v_{w}}{R(t)}\right)\left(1-\frac{R_{ev}^{3}}{R^{3}(t)}\right)\left(1-\frac{RH}{\gamma }\right),$$ (3)

where $\psi_{c}$ is a radius-dependent factor that accounts for evaporation cooling (as described below), Φ is the momentary volume fraction of solutes, γ is the water activity coefficient that accounts for non-ideal effects due to water–solute and solute–solute interactions (we assume here that γ does not depend on the water concentration), $D_{w}$ is the water diffusion constant in air, $c_{g}$ is the saturated water vapor concentration, $v_{w}$ is the liquid water molecular volume, RH is the relative humidity, and $R_{ev}$ is the equilibrium droplet radius reached by evaporation. Neglecting the possibility of crust formation due to phase separation at the droplet surface (which will be discussed later), the equilibrium droplet radius reads (see supplementary material Sec. A.4)

$$R_{ev}=R_{0}{\left(\frac{\Phi_{0}}{1-\frac{RH}{\gamma }}\right)}^{1/3},$$ (4)

with $R_{0}$ and $\Phi_{0}$ being the initial droplet radius and the initial volume fraction of solutes, respectively. The cooling factor $\psi_{c}$ in Eq. (3), which describes the slowing down of the evaporation process due to evaporation cooling, is given by (see supplementary material Sec. A.3)

$$\psi_{c}\left[\Phi \left(R\right)\right]={\left(1+\gamma \varepsilon_{c}\varepsilon_{T}\left(1-\Phi \left(R\right)\right)\right)}^{-1},$$ (5)

where $\Phi \left(R\right)=\Phi_{0}R_{0}^{3}/R^{3}$ is the momentary volume fraction of solutes, $\varepsilon_{C}=0.032\;K^{-1}$ is a numerical coefficient that accounts for the reduction of the water vapor concentration at the droplet surface due to the temperature depression [see Eq. (A19)] and $\varepsilon_{T}\equiv \frac{D_{w}c_{g}h_{ev}}{\lambda_{\mathrm{air}}}$, with $h_{ev}$ being the molecular evaporation enthalpy of water and $\lambda_{\mathrm{air}}$ the heat conductivity of air, controls the dependence of the temperature reduction at the droplet surface on the relative humidity and the solute volume fraction [see Eq. (A22)]. In the absence of solutes, Eq. (5) gives rise to a constant cooling factor, ${\psi_{c}^{\Phi_{0}\to 0}=\left(1+\gamma \varepsilon_{c}\varepsilon_{T}\right)}^{-1}$. At a room temperature of 25 °C, one obtains $\varepsilon_{T}=54$ (see the Nomenclature) and, thus, $\psi_{c}^{\Phi_{0}\to 0}\approx 0.36$, demonstrating that evaporation cooling considerably slows down the evaporation process of a pure water droplet.

To make the differential equation (3) analytically solvable, one can neglect the effect of solutes on the evaporation-cooling factor, which corresponds to replacing $\psi_{c}\left[\Phi \left(R\right)\right]$ by $\psi_{c}^{\Phi_{0}\to 0}$ in Eq. (3). From an asymptotic analysis of the solution of the differential equation (see supplementary material Sec. A.4 for more details), the radius-dependent evaporation time $t\left(R\right)$, i.e., the time it takes for the droplet radius to decrease from its initial value $R_{0}$ to R, can be written as

$$t\left(R\right)=\frac{R_{0}^{2}}{\theta \left(1-\frac{RH}{\gamma }\right)}\left[1-\frac{R^{2}}{R_{0}^{2}}-\frac{2R_{ev}^{2}}{3R_{0}^{2}}\ln\left(\frac{R_{0}\left(R-R_{ev}\right)}{R(R_{0}-R_{ev})}\right)\right],$$ (6)

with $\theta =2\gamma D_{w}v_{w}c_{g}{\left(1+\gamma \varepsilon_{c}\varepsilon_{T}\right)}^{-1}$ being a prefactor with units of a diffusion constant. We will show later that for droplets with rather low initial solute volume fractions, the solute-concentration dependence of evaporation cooling has only a minor effect on the droplet evaporation time and thus the approximation provided by Eq. (6) is rather accurate.

**FIG. 2.**
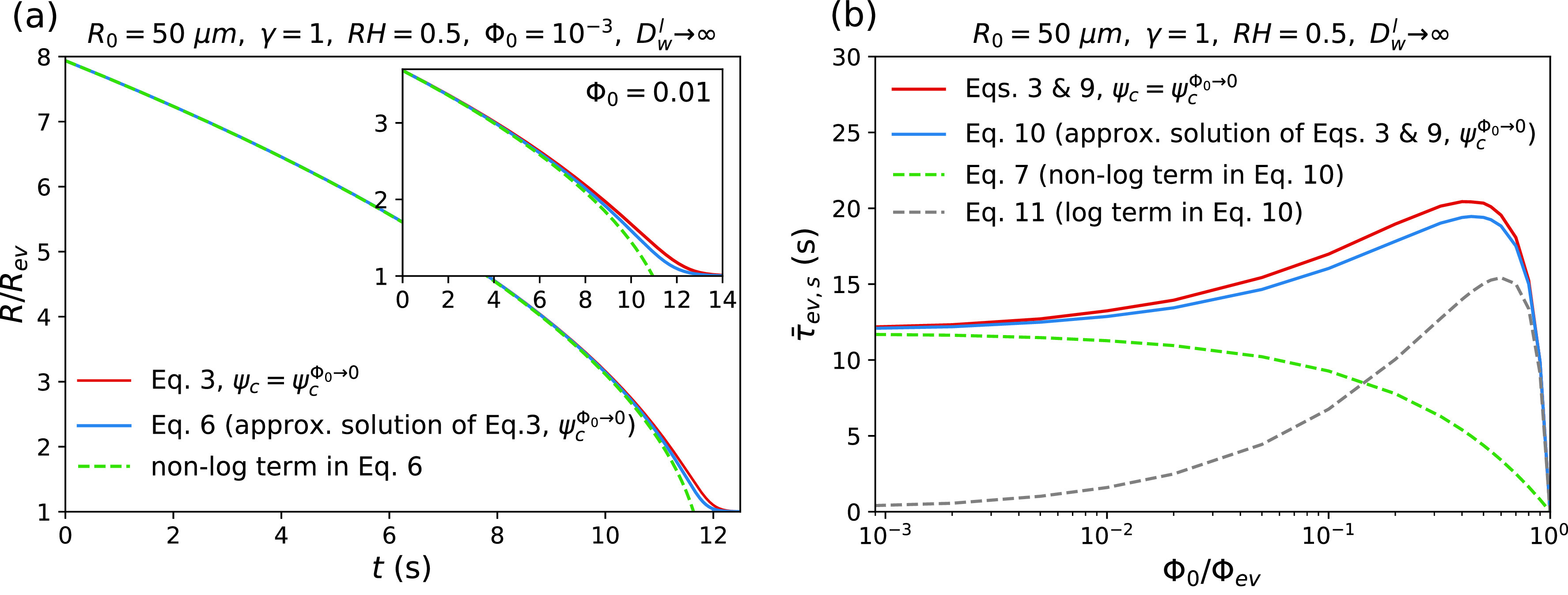
Effect of the solute-induced water vapor-pressure reduction on the drying process in the limit of an infinitely large internal water diffusion constant $D_{w}^{l}\to \infty$. The solute concentration dependence of evaporation cooling is neglected (i.e., the results are obtained using $\psi_{c}=\psi_{c}^{\Phi_{0}\to 0}$), the liquid solution is assumed ideal (γ=1), and data are shown for initial radius $R_{0}=50\;\mu m$ and relative humidity RH=0.5. Panel (a) shows the variation of the droplet radius R with time t for two different initial solute volume fractions of $\Phi_{0}=0.001$ (main figure) and $\Phi_{0}=0.01$ (inset). The solid red line indicates the results obtained from the numerical solution of Eq. (3). The solid blue and the broken green lines indicate the results obtained from Eq. (6), which is an approximate solution of Eq. (3), with and without considering the logarithmic term that reflects the solute-induced water vapor-pressure reduction. Panel (b) shows the evaporation time ${\bar{\tau }}_{ev,s}$ as a function of the initial solute volume fraction $\Phi_{0}$. The solid red line indicates the results calculated from the numerical solution of Eqs. (3) and (9). The solid blue line shows the results from Eq. (10), which is an approximate solution of Eqs. (3) and (9). Green and gray broken lines indicate the non-logarithmic and the logarithmic contributions to the evaporation time, which are obtained from Eqs. (7) and (11), respectively.

The logarithmic term in Eq. (6) reflects the osmotic slowing down of the droplet evaporation due to the solute-induced water vapor-pressure reduction. Figure 2(a), which shows $t\left(R\right)$ with and without considering the logarithmic term, reveals that this osmotic effect only becomes relevant for droplet radii close to the final equilibrium radius $R_{ev}$, where the droplet has lost most of its water content and solute effects cause a diverging evaporation time. This effect is, however, negligible for droplets with low initial solute volume fractions [see Fig. 2(a) and inset]. In this case, one can safely neglect the logarithmic term in Eq. (6), which results in the classical *R*^2^-law that is defined by $R^{2}=R_{0}^{2}-\kappa t,$^65^ with κ being the shrinkage rate of the droplet surface, which according to Eq. (6) equals $\theta (1-\frac{RH}{\gamma })$. The approximate expression for the droplet evaporation time without the logarithmic term and using $R=R_{ev}$ in Eq. (6) results in the evaporation time

$${\bar{\tau }}_{ev,s}^{\mathrm{non}-\log}=\tau_{ev}\left(1-\frac{R_{ev}^{2}}{R_{0}^{2}}\right),$$ (7)

where $\tau_{ev}$ in Eq. (7) is given by

$$\tau_{ev}=\frac{R_{0}^{2}}{\theta \left(1-\frac{RH}{\gamma }\right)},$$ (8)

and for ideal solutions (γ=1), i.e., when the solute volume fraction is negligible, represents the droplet evaporation time in the absence of solutes.^6^ The bar in Eq. (7) indicates that this equation is obtained assuming that the internal concentration profile remains homogeneous throughout the evaporation process.

As discussed above, Eq. (7) neglects a few important details of the evaporation process, such as the solute-induced water vapor-pressure reduction and the effect of solutes on evaporation cooling. The presence of an internal water concentration gradient, which arises from the finite diffusivity of water and solutes inside the droplet, is also neglected. Not only can this concentration gradient affect the droplet evaporation rate, it can also lead to formation of a solid crust on the droplet surface, which affects both the evaporation rate and the equilibrium droplet radius. The importance of these factors is evaluated next.

#### 1. Effect of the water vapor-pressure reduction

To account for the solute-induced water–vapor pressure reduction, the logarithmic term in Eq. (6) is now included. Since near the evaporation equilibrium, the droplet radius varies very slowly with time, as follows from the logarithmic term in Eq. (6) [see Fig. 2(a)], we can no longer use the definition for the evaporation time that leads to Eq. (7) (i.e., the time at which $R=R_{ev}$). Instead, we define the evaporation time as the time at which the equilibrium droplet radius $R_{ev}$ reaches 99% of the droplet radius R

$$\tau_{ev,s}=t\left(\frac{R_{ev}}{0.99}\right).$$ (9)

From Eq. (6) and considering the definition provided by Eq. (9), the evaporation time of a solute-containing droplet follows as

$${\bar{\tau }}_{ev,s}={\bar{\tau }}_{ev,s}^{\mathrm{non}-\log}+{\bar{\tau }}_{ev,s}^{\log}=\tau_{ev}\left(1-\frac{R_{ev}^{2}}{R_{0}^{2}}\right)-\frac{2R_{ev}^{2}}{3R_{0}^{2}}\tau_{ev}\ln\left(\frac{0.01}{1-\frac{R_{ev}}{R_{0}}}\right)=\tau_{ev}\left(1+\frac{2R_{ev}^{2}}{3R_{0}^{2}}\left(3.105+\ln\left(1-\frac{R_{ev}}{R_{0}}\right)\right)\right).$$ (10)

Again, the bar on top of symbols indicates the assumption that the water concentration inside the droplet remains homogeneous throughout the evaporation process (evaporation-induced concentration gradients inside the droplet are discussed later). Also, the logarithmic term ${\bar{\tau }}_{ev,s}^{\log}$ in Eq. (10) reflects the increase in the evaporation time due to the solute-induced water vapor-pressure reduction, which causes a distinct deviation from the *R*^2^-law,

$${\bar{\tau }}_{ev,s}^{\log}=\frac{2R_{ev}^{2}}{3R_{0}^{2}}\tau_{ev}\left(4.605+\ln\left(1-\frac{R_{ev}}{R_{0}}\right)\right).$$ (11)

According to Fig. 2(b), the droplet evaporation time ${\bar{\tau }}_{ev,s}$ [see Eq. (10)] and its logarithmic contribution ${\bar{\tau }}_{ev,s}^{\log}$ [see Eq. (11)] both exhibit non-monotonic variations as a function of the initial solute volume fraction $\Phi_{0}$, while the non-logarithmic term ${\bar{\tau }}_{ev,s}^{\mathrm{non}-\log}$ [see Eq. (7)] decreases with increasing $\Phi_{0}$. The reason for this can be traced to solute effects on ${\bar{\tau }}_{ev,s}^{\mathrm{non}-\log}$ and ${\bar{\tau }}_{ev,s}^{\log}$. As follows from Eq. (4), an increase in $\Phi_{0}$ increases the droplet equilibrium radius $R_{ev}$, i.e., the droplet radius at which the evaporation process stops, which tends to decrease both ${\bar{\tau }}_{ev,s}^{\mathrm{non}-\log}$ and ${\bar{\tau }}_{ev,s}^{\log}$. An increase in $\Phi_{0}$ additionally intensifies the solute-induced divergence of the logarithmic term in the function t(R) given by Eq. (6) [see Fig. 2(a) and inset], which tends to increase ${\bar{\tau }}_{ev,s}^{\log}$. Figure 2(b) reveals that at low values of $\Phi_{0}$, the second effect is dominant and thus both ${\bar{\tau }}_{ev,s}^{\log}$ and ${\bar{\tau }}_{ev,s}$ increase with increasing $\Phi_{0}$. At higher values of $\Phi_{0}$, however, the first effect becomes dominant and, consequently, ${\bar{\tau }}_{ev,s}$ and ${\bar{\tau }}_{ev,s}^{\log}$ begin to decrease with increasing $\Phi_{0}$. By inserting $R_{ev}$ from Eq. (4) into Eq. (10) and differentiating the resulting equation with respect to $\Phi_{0}$, the value of $\Phi_{0}$ at which the evaporation time is maximal is obtained as

$$\Phi_{0}^{\tau_{\text{max}}}≃0.45\left(1-\frac{RH}{\gamma }\right)=0.45{\;\Phi }_{ev},$$ (12)

where $\Phi_{ev}=1-RH/\gamma$ is the volume fraction of solutes at evaporation equilibrium, i.e., when the evaporation flux [given by Eq. (A24)] vanishes. Accordingly, when the solute volume fraction inside the droplet is initially around 45% of its final equilibrium value, the droplet experiences its maximally possible evaporation time, which according to Eqs. (4) and (10) is around 1.84 times longer than the evaporation time of a pure water droplet with the same initial radius.

Figure 2(b) also indicates that at low values of $\Phi_{0}$, $\tau_{ev,\mathrm{sol}}^{\log}$ is negligible compared to $\tau_{ev,\mathrm{sol}}^{\mathrm{non}-\log}$, meaning that the *R*^2^-law is almost valid and thus Eq. (7) is accurate. In the case of droplets with high initial solute volume fractions, however, the logarithmic terms in Eqs. (6) and (10) are important and can no longer be neglected. By equating Eqs. (7) and (11), one finds that the effect of the water vapor-pressure reduction becomes dominant when $\Phi_{0}$ becomes higher than approximately $0.13\Phi_{ev}$.

#### 2. Effect of nonvolatile solutes on evaporation cooling

The evaporation-induced cooling of the droplet decreases the temperature at the droplet surface compared to the ambient air by $\Delta T=T_{s}-T_{0}$, where $T_{s}$ and $T_{0}$ are the temperatures at the droplet surface and far away from the droplet, respectively. The value of ΔT is obtained by solving the coupled heat-conduction and water–vapor-diffusion equations outside the droplet (see supplementary material Sec. A.3) as

$$\Delta T=\gamma \varepsilon_{T}\psi_{c}\left[\Phi \left(R\right)\right]\left(1-\Phi \left(R\right)-\frac{RH}{\gamma }\right),$$ (13)

where $\psi_{c}\left[\Phi \left(R\right)\right]$ is the cooling factor given by Eq. (5). Equation (13) reveals that not only ΔT is linearly related to the ambient relative humidity RH, as already reported in the absence of solutes,^6^ but it also depends on the momentary volume fraction of solutes Φ, which increases with time due to the water loss by evaporation. This equation shows that at evaporation equilibrium, i.e., when the momentary volume fraction of solutes reaches its equilibrium value, $\Phi_{\mathrm{ev}}=1-RH/\gamma$, ΔT goes to zero, meaning that the droplet reaches thermal equilibrium with its environment as well.

**FIG. 3.**
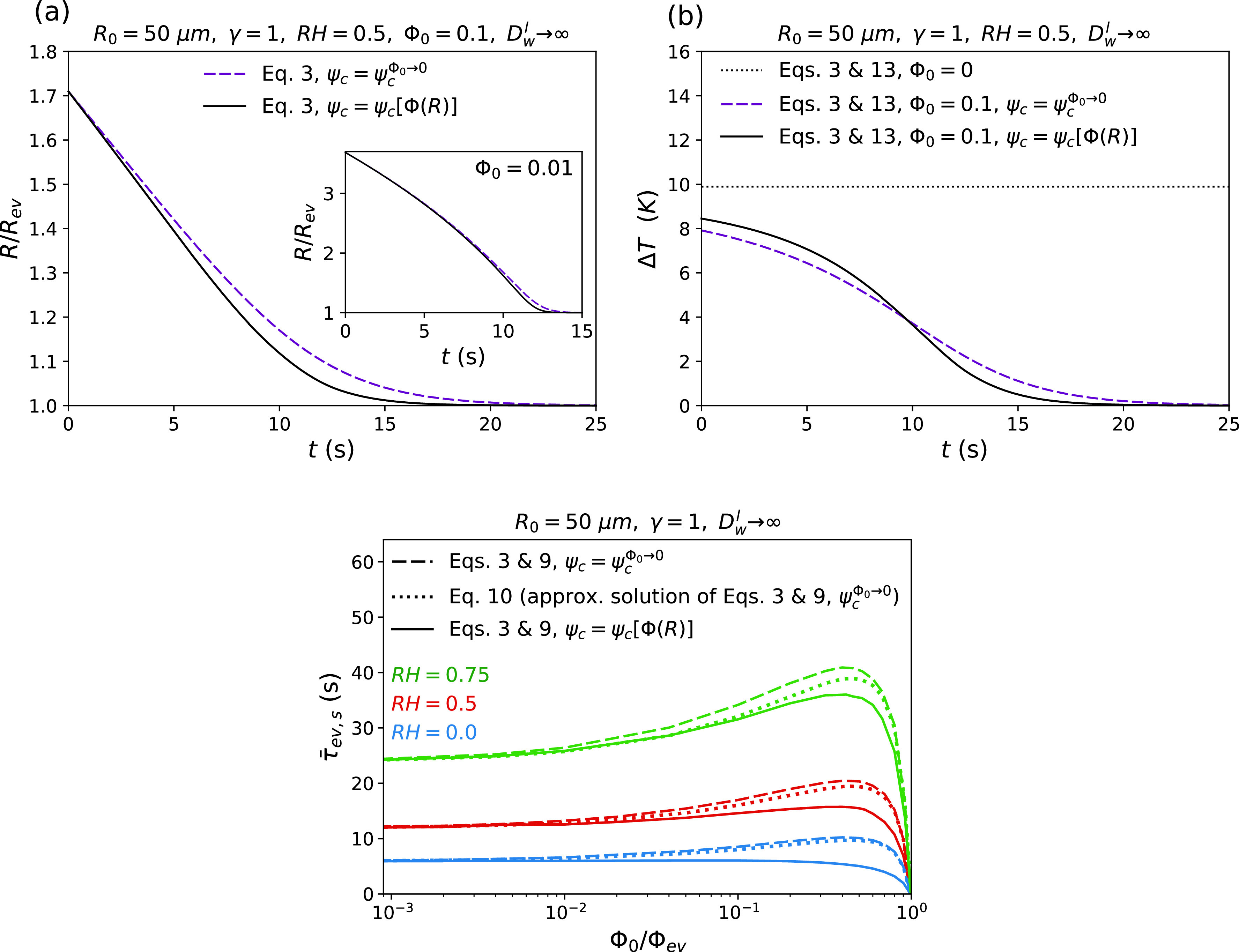
Effect of nonvolatile solutes on evaporation cooling and, consequently, on the evaporation time in the limit of infinitely high internal water diffusivity $D_{w}^{l}\to \infty$. (a) Variation of the droplet radius R with time t for RH=0.5 and two different initial solute volume fractions of $\Phi_{0}=0.1$ (main figure) and $\Phi_{0}=0.01$ (inset). Solid and broken lines indicate the results from the numerical solutions of Eq. (3), with and without considering the solute-concentration dependence of evaporation cooling {corresponding to using $\psi_{c}=\psi_{c}[\Phi \left(R\right)]$ and $\psi_{c}=\psi_{c}^{\Phi_{0}\to 0}$ in the calculations}. (b) Variation of the evaporation-induced temperature reduction at the droplet surface, ΔT, with time t. The results are obtained from Eqs. (3) and (13) for fixed relative humidity of RH=0.5. The dotted line indicates the results for a pure water droplet, $\Phi_{0}=0$. Solid and broken lines indicate the results for a solute-containing droplet with $\Phi_{0}=0.1$, with and without considering the solute-concentration dependence of evaporation cooling. (c) Evaporation time of a solute-containing droplet ${\bar{\tau }}_{ev,s}$ as a function of the initial solute volume fraction $\Phi_{0}$ for different relative humidities. Solid and broken lines indicate the results obtained from numerical solutions of Eqs. (3) and (9), with and without considering the solute-concentration dependence of evaporation cooling, respectively. Dotted lines show Eq. (10), which is an approximate solution of Eqs. (3) and (9) that neglects the effect of solutes on evaporation cooling.

So far, we used Eq. (10) to calculate the radius-dependent evaporation time. As mentioned before, this equation neglects the effect of solutes on evaporation cooling, which corresponds to using the cooling factor in the absence of solutes, $\psi_{c}^{\Phi_{0}\to 0}$, in the calculations. According to Eq. (13), this approximation results in a linear decrease in ΔT with an increase in the momentary solute volume fraction Φ. The solute-concentration dependence of the evaporation-cooling factor, however, causes a non-linear relationship between ΔT and Φ, as follows from Eqs. (5) and (13). To account for this effect, one has to calculate the shrinkage rate of the droplet radius directly from Eq. (3), which has to be done numerically. Figure 3(a) shows the time-dependent droplet radii calculated from the numerical solution of Eq. (3), with and without considering the effect of solutes on evaporation cooling {corresponding to using $\psi_{c}\left[\Phi \left(R\right)\right]$ and $\psi_{c}^{\Phi_{0}\to 0}$ in the calculations, respectively}. This figure indicates that the solute-concentration dependence of evaporation cooling slightly speeds up the evaporation process. This speed up becomes more significant as the evaporation proceeds, which can be attributed to the increased solute concentration within the droplet. This effect is found to be more pronounced for droplets with higher initial solute volume fractions [see Fig. 3(a) and inset].

Figure 3(b) shows the time-dependent variation of the evaporation-induced temperature reduction at the droplet surface ΔT in the absence of solutes, and in the presence of solutes with and without considering solute effects on evaporation cooling. This figure shows that the temperature reduction at the surface of a pure water droplet remains constant throughout the evaporation process. In the presence of solutes, however, the temperature reduction decreases with time and eventually reaches zero at evaporation equilibrium. Figure 3(b) reveals that neglecting the effect of solutes on evaporation cooling slightly reduces the time-dependent decay of ΔT, which causes a delay in the evaporation equilibrium state.

To better understand how the solute-concentration dependence of evaporation cooling affects the droplet evaporation time, the evaporation time is now calculated from the numerical solution of Eq. (3), using the definition provided by Eq. (9). The results obtained from the numerical calculations with and without considering the solute-concentration dependence of evaporation cooling {which correspond to using $\psi_{c}\left[\Phi \left(R\right)\right]$ and $\psi_{c}^{\Phi_{0}\to 0}$ in the calculations} are, respectively, shown by solid and broken lines in Fig. 3(c). Also, dotted lines in this figure show the results obtained from Eq. (10), which is an approximate analytical solution of Eq. (3) when the effect of solutes on evaporation cooling is neglected. This figure clearly shows that the solute-concentration dependence of evaporation cooling causes a decrease in the droplet evaporation time, especially when $\Phi_{0}$ becomes close to $\Phi_{0}^{\tau_{\;\text{max}}}$ [see Eq. (12)]. The approximation used in the derivation of Eq. (10) [which is described by Eq. (A32)] is, however, found to partly compensate for the difference caused by neglecting such concentration dependence, especially at high relative humidity. According to this figure, at low initial solute volume fractions $\Phi_{0}\leq 0.01\Phi_{\mathrm{ev}}$, which for RH≤0.5 includes the normal solute volume fraction of saliva, $\Phi_{0}≃0.05,$^77^ one can safely use Eq. (10) to estimate the droplet evaporation time. At higher values of $\Phi_{0}$, however, this approximation starts to become inaccurate. Figure 3(c) also indicates that the effect of the solute-concentration dependence of evaporation cooling is more significant in dry environments.

### B. Droplet evaporation in the presence of nonvolatile solutes considering evaporation-induced internal concentration gradients

In the previous section, the particle diffusivity inside the droplet was assumed to be sufficiently rapid, so that the solute remains evenly distributed. In the fast evaporation scenario (see Fig. 1), however, the droplet exhibits an increased solute concentration at its surface, which results in a water concentration gradient inside the droplet. To account for this concentration gradient, one needs to solve the diffusion equation [Eq. (1)] not only in the vapor phase outside the droplet, but also in the liquid phase for both water and solutes. As discussed in supplementary material Sec. B.1, the diffusion equations for solvent and solutes in an incompressible fluid mixture are simultaneously solvable only if the diffusion coefficients of all components in the liquid mixture are equal. Here, we use this assumption, which approximates the behavior of fast-diffusing solutes rather accurately. Accordingly, the liquid water diffusion coefficient $D_{w}^{l}$ is used to calculate the concentration profile inside the droplet. We will also show results for diffusion coefficients smaller than $D_{w}^{l}$, which is an approximate way of treating the effect of slowly diffusing solutes, as discussed before.

**FIG. 4.**
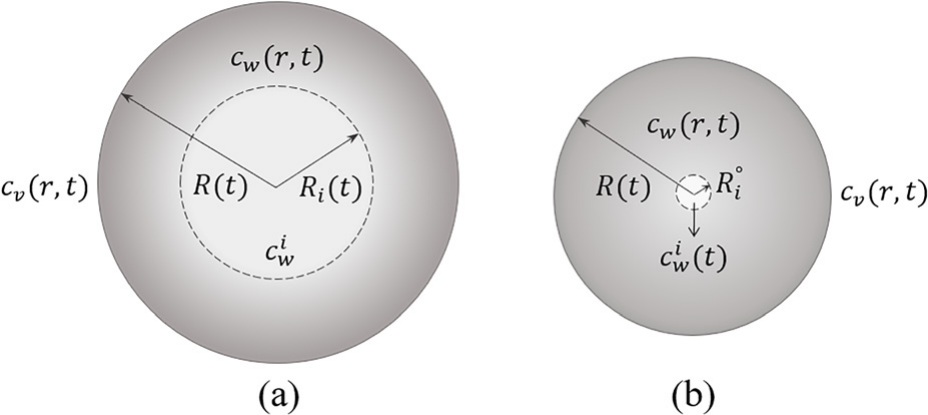
The model used to account for an evaporation-induced inhomogeneous water concentration inside the droplet. Here, the water concentration profile within the inner core of radius $R_{i}\left(t\right)$ (the bright area) is assumed to remain uniform throughout the drying process while the outer shell of radius $R\left(t\right)$ (the dark area) exhibits an inhomogeneous water concentration. This model leads to two drying stages: (a) in the first stage, the water concentration in the inner core $c_{w}^{i}$ is constant while the radius of the inner core $R_{i}$ varies with time, (b) in the second stage the radius of the inner core has a small constant value $R_{i}^{{}^{\circ}}$ (which is of molecular size) while $c_{w}^{i}$ is time-dependent.

The general problem one encounters when considering diffusion in radial coordinates is that the diffusion equation [Eq. (1)] has a singularity at r=0, at the center of the droplet. The easiest way to deal with this problem is to solve the discretized diffusion equation using numerical methods,^78^ where the singularity is removed from the solution domain. To solve the diffusion equation inside the droplet analytically, we divide the droplet volume into two regions: an inner core, where the concentration profile remains uniform, and an outer shell, where the liquid phase exhibits a concentration gradient (see Fig. 4). The water concentration profile inside the droplet can therefore be calculated from the solution of the diffusion equation in the outer shell that does not include the singular point.

Additionally, we consider a two-stage evaporation model. In the first stage [Fig. 4(a)], the droplet evaporation reduces the droplet size and, at the same time, increases the thickness of the outer shell, where the water concentration has decreased due to loss of water by evaporation and the liquid phase is inhomogeneous. In this stage, both the droplet radius R and the radius of the inner core $R_{i}$ shrink with time while the water concentration in the inner core $c_{w}^{i}$ remains equal to its initial value. When $R_{i}$ reaches a cut off value $R_{i}^{{}^{\circ}}$ (which is of molecular size), the drying process turns into the second stage [Fig. 4(b)]. In this stage, $R_{i}$ is fixed to $R_{i}^{{}^{\circ}}$ while R and $c_{w}^{i}$ are considered as time-dependent parameters, which reach their equilibrium values at the end of the second drying stage.

We first assume that the water diffusion coefficient in the liquid phase $D_{w}^{l}$ is independent of solute concentration (concentration dependence of $D_{w}^{l}$ is discussed next). Using the two-stage model described above, the liquid water density profiles in the first drying stage $c_{w}^{1st}(r)$ and in the second drying stage $c_{w}^{2nd}(r)$ are obtained as (the calculations are detailed in supplementary material Sec. B)

cw1str=1−Φ0vw r≤Ri, 1−Φ0vw+1−Φ0−RHγRr−RRivwαγ−1+RRi+αεTεc1−Φ0 Ri<r≤R, (14)

cw2ndr=1−Φ0R03R 3+μRHγvw1+μ r≤Ri°,1−Φ0R03R 3+μRHγvw1+μ+1vw1−Φ0R03R 3−RHγRr−RRi°αγ−1+RRi°1+μ+αεTεc1−Φ0R03R 3+μRHγ Ri°<r≤R. (15)

R and $R_{i}$ in the above equations are time-dependent parameters and μ in Eq. (15) is a function of R, which is given by

$$\mu =-\frac{\frac{R_{i}^{{}^{\circ}2}}{R^{2}}+2\frac{R}{R_{i}^{{}^{\circ}}}-3}{2\left(\frac{\alpha }{\gamma }-1+\frac{R}{R_{i}^{{}^{\circ}}}+\alpha \varepsilon_{T}\varepsilon_{c}\right)}.$$ (16)

The numerical prefactor α in Eqs. (14)–(16) is given by $\alpha =D_{w}^{l}/(D_{w}c_{g}v_{w})$ and describes the ratio of internal and external water diffusivities. Considering the values listed in the Nomenclature, this prefactor is approximately equal to α≈4 at 25 °C. Using the assumption of infinitely rapid diffusion in the droplet, which is the main assumption in the previous section, α goes to infinity. In this section, we aim to investigate the effect of the internal diffusion constant on the water evaporation process, and thus the calculations are done for different finite values of α.

Neglecting the probability of crust formation due to the increased solute concentration at the droplet surface (which is discussed later), one can calculate the droplet evaporation time as

$$\tau_{ev,\;s}=\tau_{ev,s}^{1st}+\tau_{ev,s}^{2nd},$$ (17)

where $\tau_{ev,s}^{1st}$ and $\tau_{ev,s}^{2nd}$ are the times of the first and the second drying stages, respectively. As detailed in supplementary material Sec. B.4, mass conservation of water and solutes gives rise to the following differential equations that describe the time-dependent variation of the droplet radius in the two drying stages,

$${\left.\frac{dR}{dt}\right|}_{1st}=-\frac{D_{w}^{l}\left(1-\Phi_{0}-\frac{RH}{\gamma }\right)}{R\left(\frac{\alpha }{\gamma }-1+\frac{R}{R_{i}}+\alpha \varepsilon_{T}\varepsilon_{c}\left(1-\Phi_{0}\right)\right)},$$ (18)

$${\left.\frac{dR}{dt}\right|}_{2nd}=-\frac{\frac{D_{w}^{l}}{R}\left(1-\frac{\Phi_{0}R_{0}^{3}}{R_{\;}^{3}}-\frac{RH}{\gamma }\right)}{\left(\frac{\alpha }{\gamma }-1+\frac{R}{R_{i}^{{}^{\circ}}}\right)\left(1+\mu \right)+\alpha \varepsilon_{T}\varepsilon_{c}\left(1-\frac{\Phi_{0}R_{0}^{3}}{R_{\;}^{3}}+\mu \frac{RH}{\gamma }\right)}.$$ (19)

**FIG. 5.**
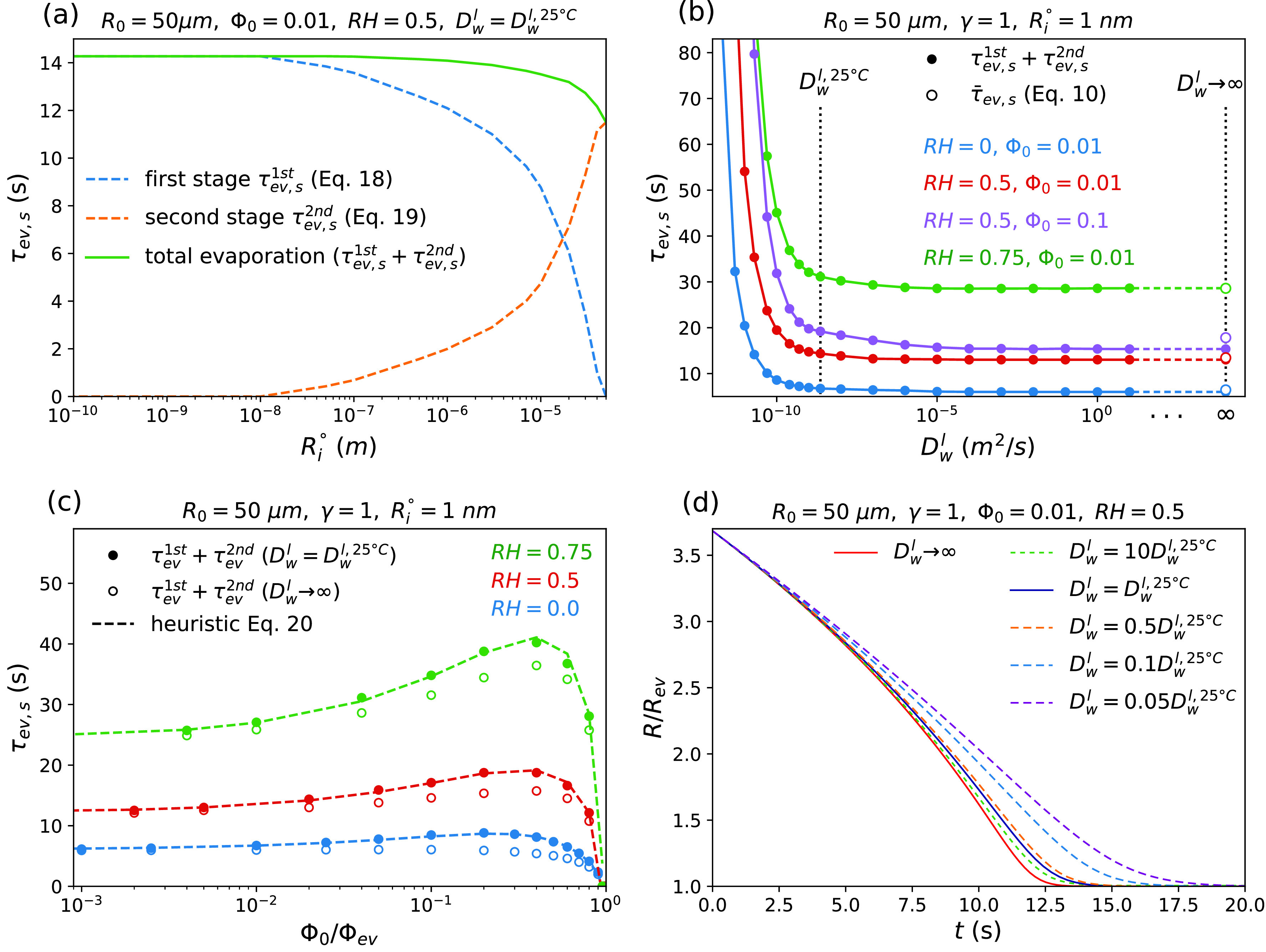
Results obtained using the two-stage evaporation model that accounts for an inhomogeneous internal water concentration. (a) The times of the two drying stages ($\tau^{1st}$ and $\tau^{2nd})$ and the total evaporation time $\tau_{ev,s}=\tau_{ev,s}^{1st}+\tau_{ev,s}^{2nd}$ as a function of $R_{i}^{{}^{\circ}}$, with $R_{i}^{{}^{\circ}}$ being the radius of the inner core $R_{i}$ in the second drying stage (see Fig. 3). $\tau^{1st}$ and $\tau^{2nd}$ are calculated from Eqs. (18) and (19), respectively, for RH=0.5, $\Phi_{0}=0.01$, and using the value of the water diffusion coefficient at 25°C, $D_{w}^{l,25{}^{\circ}C}=2.3\times 10^{-9}\;m^{2}/s$ (see the Nomenclature). (b) Evaporation time $\tau_{ev,s}$ as a function of the water diffusion coefficient in the liquid phase $D_{w}^{l}$. Solid symbols show the results from Eqs. (18) and (19) using $R_{i}^{{}^{\circ}}=1\;\mathrm{nm}$. Open symbols to the right depict the results from Eq. (10) in the limit of infinitely large internal water diffusion constant. The vertical dotted lines indicate $D_{w}^{l}=D_{w}^{l,25{}^{\circ}C}$ (see the Nomenclature) and $D_{w}^{l}\to \infty$. (c) Variation of the evaporation time $\tau_{ev,s}$ with the initial solute volume fraction $\Phi_{0}$. Solid and open symbols depict the results from Eqs. (18) and (19) (again, using $R_{i}^{{}^{\circ}}=1\;\mathrm{nm}$) for $D_{w}^{l}=D_{w}^{l,25{}^{\circ}C}$ and $D_{w}^{l}\to \infty$, respectively. Broken lines indicate the results estimated from Eq. (20) using the fit parameters $a_{\tau 1}=1.03$ and $a_{\tau 2}=5/6$. (d) Droplet radius R as a function of time t for RH=0.5, $\Phi_{0}=0.01$, and different internal water diffusion coefficients. The results for $D_{w}^{l}\to \infty$ are calculated from Eq. (3) and those for $D_{w}^{l}<\infty$ are obtained from Eq. (18).

Equations (18) and (19) are solved using the numerical approaches explained in supplementary material Sec. B.4 to yield t(R) in each drying stage and, consequently, $\tau_{ev,s}^{1st}$ and $\tau_{ev,s}^{2nd}$. In our calculations, the radius of the inner core at which the droplet turns into the second drying stage $R_{i}^{{}^{\circ}}$ is set to 1 nm, which is small enough so that the calculated evaporation time is independent of $R_{i}^{{}^{\circ}}$ [see Fig. 5(a)]. Figure 5(b) shows the droplet evaporation time obtained from Eqs. (17)–(19) as a function of the liquid water diffusion coefficient $D_{w}^{l}$. This figure demonstrates that a decrease in $D_{w}^{l}$ increases the droplet evaporation time. This is consistent with experimental evidence indicating that the extremely extended evaporation time of secondary organic aerosol (SOA) particles can be attributed to the reduced water diffusivity in the viscous phase.^33,79^ For a high liquid water diffusion coefficient, however, $\tau_{ev,s}$ is found to be independent of $D_{w}^{l}$. Figure 5(b) also confirms that for the rather low initial solute volume fraction of $\Phi_{0}=0.01$, Eq. (10) accurately predicts the evaporation time in the limit of $D_{w}^{l}\to \infty$, as discussed in Sec. II A 2.

As stated before, our diffusion model is valid only when we assume that the diffusion coefficients of water and solutes in the liquid phase are equal, $D_{s}^{l}=D_{w}^{l}$ (see supplementary material Sec. B.1 for more details). Using this assumption and neglecting the concentration dependence of the diffusion coefficient, which will be discussed next, the value reported in the Nomenclature for the water diffusion coefficient in pure water at 25 °C, $D_{w}^{l,25\;{}^{\circ}C}=2.3\times 10^{-9}\;m^{2}/s$, which is indicated by the dotted vertical line in Fig. 5(b), can be used to estimate the evaporation time of a solute-containing water droplet at 25 °C. However, most relevant solutes diffuse slower than water molecules (i.e., $D_{s}^{l}<D_{w}^{l}$), and thus the effective diffusion coefficient to be used inside the droplet is the solute diffusion coefficient, as discussed before. According to Fig. 5(a), therefore, one expects the evaporation process of droplets that contain slowly diffusing solutes to take longer than what is estimated here using the water diffusion coefficient in the calculations.

Figure 5(c) shows the evaporation times obtained from Eqs. (17)–(19) using the water diffusion coefficient at 25 °C, $D_{w}^{l}=D_{w}^{l,25\;{}^{\circ}C},$ together with results obtained in the limit of $D_{w}^{l}\to \infty$, which leads to a homogeneous internal water concentration. According to this figure, although the effect of internal concentration gradients on the droplet evaporation time is negligible at low initial solute volume fractions, neglecting this effect can make a relatively large error at higher values of $\Phi_{0}$, especially when $\Phi_{0}$ becomes close to $\Phi_{0}^{\tau_{\text{max}}}$ [see Eq. (12)]. The relative error is found to be more significant in dry environments because the drier the air, the faster the water evaporates from the droplet surface and the larger the water concentration gradient becomes. This figure also shows that the presence of internal concentration gradients cannot significantly change the value of the solute volume fraction at which the droplet evaporation time is maximal, denoted as $\Phi_{0}^{\tau_{\text{max}}}$.

As mentioned before, the differential equations (18) and (19) that are used here to calculate the evaporation time are not analytically solvable and, hence, the evaporation time is calculated using numerical methods. To provide an equation for estimating $\tau_{ev,s}$ as a function of the relevant parameters that characterize the mechanisms discussed so far (i.e., solute-induced water vapor-pressure reduction, concentration dependence of the evaporation-cooling effect, and evaporation-induced concentration gradients inside the droplet), we construct a heuristic fit function inspired by Eq. (10),

$$\tau_{ev,s}=a_{\tau 1}\frac{R_{0}^{2}}{\theta^{\prime }\left(1-\frac{RH}{\gamma }\right)}\left[1+a_{\tau 2}{\left(\frac{R_{ev}}{R_{0}}\right)}^{2}\left(3.105+\ln\left(1-\frac{R_{ev}}{R_{0}}\right)\right)\right],$$ (20)

where $a_{\tau 1}$ and $a_{\tau 2}$ are fit parameters and $\theta^{\prime }$ is a modified numerical prefactor given by

$$\theta^{\prime }=2\gamma D_{w}c_{g}v_{w}{\left(1+\gamma \varepsilon_{C}\varepsilon_{T}\left(1-\Phi_{0}\right)\right)}^{-1}.$$ (21)

Figure 5(c) demonstrates that Eq. (20) (shown by broken lines) with fit parameters $a_{\tau 1}=1.03$ and $a_{\tau 2}=\frac{5}{6}$ perfectly fits the data over a wide range of $\Phi_{0}$ and RH. It is worth mentioning that the values used for $a_{\tau 1}$ and $a_{\tau 2}$ are the averages of the fit parameters obtained for different values of $\Phi_{0}$, RH, and γ.

Finally, the effect of an internal concentration gradient on the time-dependent variation of the droplet radius is investigated. The point to note here is that in the case of a droplet with a finite internal water diffusivity, the first drying stage is dominant. Indeed, when $R_{i}^{{}^{\circ}}$ is set to a sufficiently small value (on the order of a few nano-meters or smaller), the time of the second drying stage $\tau_{ev,s}^{2nd}$ goes to zero [as demonstrated in Fig. 5(a)], meaning that the second drying stage becomes negligible. Therefore, the time-dependent radius of a droplet with finite $D_{w}^{l}$ can be calculated from Eq. (18) only, which describes the first drying stage. Also, R(t) in the limit of $D_{w}^{l}\to \infty$ can be obtained from Eq. (3), which neglects the internal water concentration gradients. The results are shown in Fig. 5(d). This figure indicates that in the beginning of the evaporation process, when the evaporation-induced concentration gradients are not significant yet, the shrinkage rate of the droplet radius is almost independent of the internal water diffusion coefficient. As the evaporation proceeds, however, this parameter becomes important and affects the droplet evaporation time. This figure also shows that in the presence of slowly diffusing solutes (i.e., solutes with lower diffusion coefficients than water), the effect of the evaporation-induced internal water concentration gradient is more significant, as expected.

#### 1. Effect of the solute-concentration dependence of the water diffusivity inside the droplet

The diffusion coefficient of water is known^43^ to decrease in the presence of strongly hydrated solutes, such as NaCl, NaI, and NaBr. In contrast, the presence of weakly hydrated solutes, such as KCl, KBr, and KI, increases the water self-diffusion coefficient, presumably by disrupting hydrogen bonding. According to Fig. 5(b), such variations in the water diffusion coefficient can slow down or speed up the droplet evaporation process. Therefore, the evaporation model introduced in Sec. II B must be modified in order to account for the solute-concentration dependence of water diffusivity inside the droplet. Here, we assume that the internal water diffusion coefficient is linearly dependent on the solute concentration, which approximates the behavior of NaCl and NaBr salts rather accurately.^43^ We assume such a linear dependence also for other solutes such as proteins, carbohydrates, and viruses. Accordingly, we write

$$D_{w}^{\mathrm{sol}}\left(r,t\right)=D_{w}^{l}\left(1-\beta c_{s}\left(r,t\right)\right)=D_{w}^{l}\left(1-\frac{\beta }{v_{s}}\left(1-v_{w}c_{w}\left(r,t\right)\right)\right),$$ (22)

with $D_{w}^{\mathrm{sol}}$ and $D_{w}^{l}$ being the water diffusion coefficients in an aqueous solution and in pure water, respectively, β being a solute-specific constant ($∼0.065\;M^{-1}$ for NaCl and $∼0.058\;M^{-1}$ for NaBr solutions^43^), $v_{s}$ being the molecular volume of solutes, and $c_{s}\left(r,t\right)=1/v_{s}-v_{w}c_{w}\left(r,t\right)/v_{s}$ being the solute concentration profile.

According to Eq. (22), when the solute concentration at the droplet surface reaches a critical value of $c_{s}^{*}=\beta^{-1}$, the water diffusion coefficient at the droplet surface $D_{w}^{\mathrm{sol}}(R)$ reaches zero. Therefore, in the cases where $c_{s}^{*}$ is lower than the equilibrium concentration of solutes $c_{s}^{ev}=(1-RH/\gamma )/N_{A}v_{s}$, i.e., the solute concentration at which the droplet reaches the evaporation-equilibrium state [see Eq. (A14)], the water diffusion flux at the droplet surface $-D_{w}^{\mathrm{sol}}(R){\left.\frac{\partial c_{l}}{\partial r}\right|}_{r=R}$ goes to zero before the net evaporation flux $k_{e}c_{w}\left(R\right)-k_{c}c_{v}(R)$ vanishes. In such case, the reactive boundary condition given by Eq. (A3) cannot be satisfied, which prevents the numerical solution of the diffusion equation from converging. To avoid this problem, we exclusively consider the parameter range of β and RH so that $c_{s}^{ev}\leq c_{s}^{*}$. In this limit, the water diffusion coefficient remains non-zero during the entire drying process and minimally reaches zero in the evaporation-equilibrium state. At first, the possibility of phase separation at high solute concentrations is neglected. In reality, when the solute concentration at the droplet surface exceeds a critical supersaturation concentration $c_{s}^{\text{max}},$^80^ which is normally reached before $D_{w}^{\mathrm{sol}}$ goes to zero, a solid crust forms at the droplet surface. For example, $c_{s}^{\text{max}}$ in the case of aqueous NaCl droplets at 20 °C is around 11.1 M,^8^ and β for a NaCl solution is $∼0.065\;M^{-1}.$^43^ Therefore, according to Eq. (22), the water diffusion constant in a liquid droplet of NaCl solution at the onset of crust formation is ∼28% of the water diffusion constant in pure water. The possibility of crust formation is accounted for in Sec. II B 2.

**FIG. 6.**
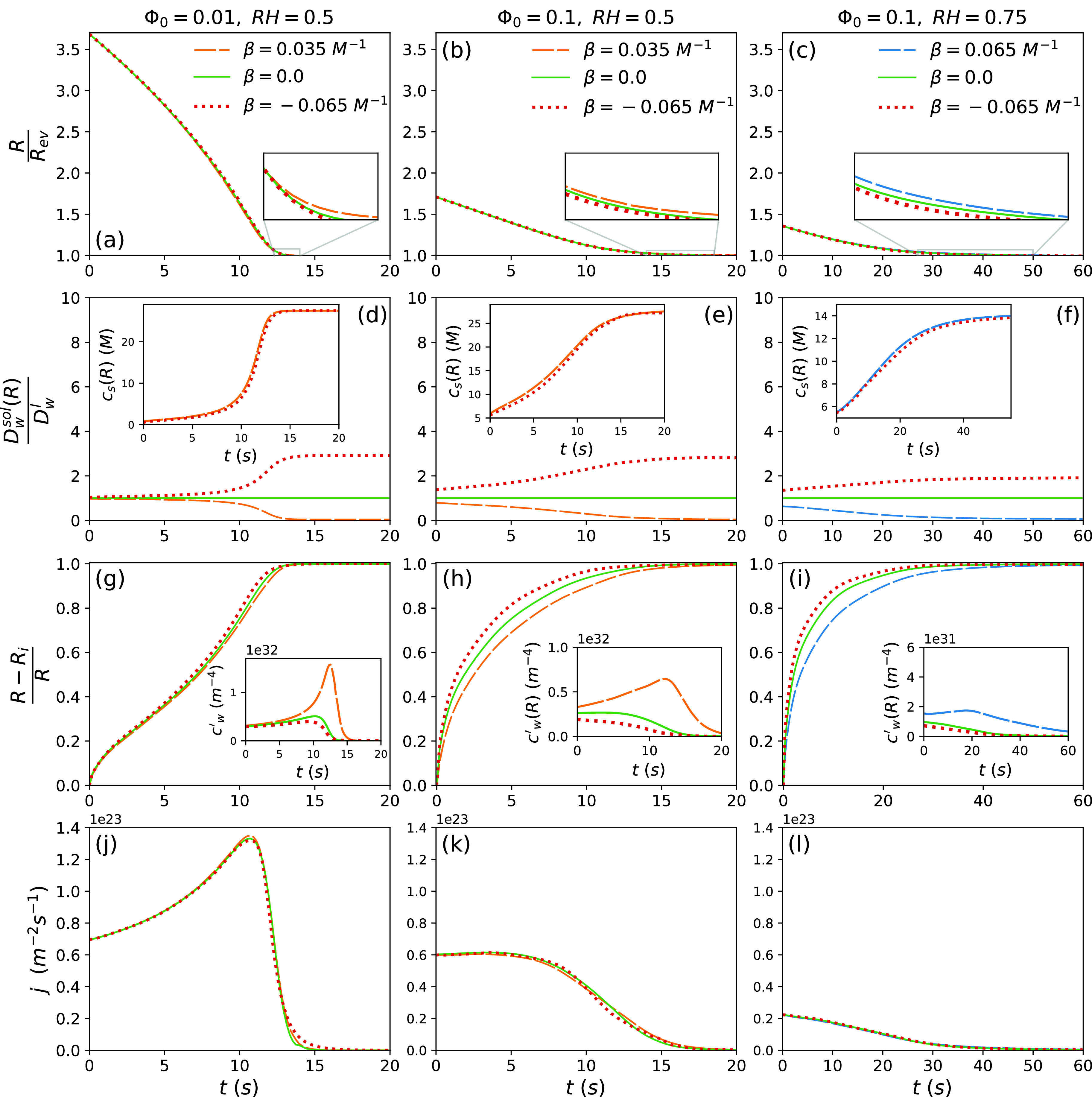
Results obtained taking into account the solute-concentration dependence of the internal water diffusivity [see Eq. (22)]. This figure shows the time-dependent variation of [(a)–(c)] the droplet radius R, [(d)–(f)] the water diffusion constant at the droplet surface $D_{w}^{\mathrm{sol}}(R)$, [(d)–(f), insets] the surface concentration of solutes $c_{s}(R)$, [(g)–(i)] the thickness of the outer droplet shell $R-R_{i}$, [(g)–(i), insets] the water concentration gradient at the droplet surface $c_{w}^{\prime }\left(R\right)=-{\left.\partial c_{w}/\partial r\right|}_{r=R}$, and [(j)–(l)] the water flux density $j=D_{w}^{\mathrm{sol}}\left(R\right)c_{w}^{\prime }\left(R\right)$. The results are calculated using the numerical method described in supplementary material Sec. C.2, which accounts for the solute-concentration dependence of the internal water-diffusion coefficient, for $\Phi_{0}=0.01$ and RH=0.5 (first column), $\Phi_{0}=0.1$ and RH=0.5 (second column), and $\Phi_{0}=0.1$ and RH=0.75 (third column). The solute and water molecular volumes are assumed equal $v_{s}=v_{w},$ and the values reported in the Nomenclature are used for the molecular volume of water $v_{w}=3\times 10^{-29}\;m^{3}$ and the water diffusion coefficient in pure water $D_{w}^{l}=D_{w}^{l,25{}^{\circ}C}=2.3\times 10^{-9}\;m^{2}/s$.

By incorporating $D_{w}^{\mathrm{sol}}$ from Eq. (22) into the water-diffusion equation [Eq. (1)] and using the numerical methods described in supplementary material Sec. C to solve this equation along with the heat and mass conservation equations, the time-dependent radius of the evaporating droplet is determined. Panels (a)–(c) of Fig. 6 show the results in the absence of solutes and in the presence of solutes that increase or decrease the internal water diffusivity [respectively, corresponding to setting β=0, β<0, and β>0 in Eq. (22)]. This figure indicates that the solute-concentration dependence of the diffusion coefficient does not significantly change the time-dependent shrinkage of the droplet radius, even for droplet radii close to the final equilibrium radius, where the solute concentration at the droplet surface tends to its maximum value. The reason for this behavior is discussed below.

The presence of solutes inside a drying droplet has two effects on the internal water diffusivity: first, it changes the water diffusion coefficient in the droplet at the onset of evaporation, when the water concentration is homogeneous, and second, it induces a time-dependent diffusivity profile inside the droplet. Accordingly, Eq. (22) can be rewritten as

$$D_{w}^{\mathrm{sol}}\left(r,t\right)=D_{w}^{\mathrm{sol},i}+\Delta D_{w}^{\mathrm{sol}}\left(r,t\right)=D_{w}^{l}\left(1-\beta c_{s}^{i}\right)+\Delta D_{w}^{\mathrm{sol}}\left(r,t\right),$$ (23)

where $D_{w}^{\mathrm{sol},i}$ is the initial water diffusion coefficient in the liquid droplet and thereby also the water diffusion coefficient within the inner core where the solute concentration equals its initial value (see Fig. 4), $c_{s}^{i}$ is the initial solute concentration, and $\Delta D_{w}^{\mathrm{sol}}\left(r,t\right)$ indicates the time-dependent local variation of the water diffusion coefficient, which is due to the evaporation-induced concentration gradient in the outer shell (see Fig. 4). We first investigate the effect of the solute-concentration dependence of the initial water diffusivity on the droplet evaporation. According to Figs. 5(b) and 5(d), this effect can significantly change the droplet evaporation time only if the initial deviation of the internal water diffusivity from its value in pure water is sufficiently large ($\frac{D_{w}^{\mathrm{sol},i}}{D_{w}^{l}}<0.5$ or $\frac{D_{w}^{\mathrm{sol},i}}{D_{w}^{l}}>10$). For the parameter range studied here, however, this deviation is rather small [see Fig. 6, panels (d)–(f)], and thus the evaporation process is not affected by the solute-concentration dependence of $D_{w}^{\mathrm{sol},i}$ [see Fig. 6, panels (a)–(c) and (j)–(l)]. This is expected to be the case for most droplets of ionic solutions, where β is typically small, and for most respiratory droplets, where the initial solute concentration $c_{s}^{i}$ is not large enough to significantly affect $D_{w}^{\mathrm{sol},i}$. As evaporation proceeds, the increased solute concentration at the droplet surface gradually changes the water diffusion coefficient at the surface from its initial value and thus the second effect, i.e., the effect of an inhomogeneous diffusivity profile, becomes important. Figure 6, however, shows that this effect does not significantly change the shrinkage rate of the drying droplet, even in the cases with β>0, where the water diffusion coefficient at the droplet surface decreases down to zero during the evaporation process (this effect only makes a slight change in the droplet shrinkage rate at the very end of the evaporation process, when $R≃R_{ev}$). The reason for this is examined next.

As follows from Eq. (22), an increase in β decreases the internal water diffusion coefficient at the droplet surface $D_{w}^{\mathrm{sol}}(R)$ [see Fig. 6, panels (d)–(f)]. On the other hand, an increase in β slows down the internal mixing process and, consequently, reduces the growth rate of the outer shell, i.e., the shell within which the liquid phase exhibits a concentration gradient [see Fig. 6, panels (g)–(i)], while the solute concentration at the droplet surface remains almost independent of β [see Fig. 6, insets of panels (d)–(f)]. This means that the water concentration gradient at the droplet surface $c_{w}^{\prime }\left(R\right)=-{\left.\frac{\partial c_{w}}{\partial r}\right|}_{r=R}$ increases with an increase in β [see Fig. 6, insets of panels (g)–(i)]. As a result, the water flux density at the liquid–vapor interface j, which is the product of $D_{w}^{\mathrm{sol}}(R)$ and $c_{w}^{\prime }\left(R\right)$, is found to remain almost independent of β [see Fig. 6, panels (j)–(l)]. This implies that the solute-induced local variation of the internal water diffusivity has only a minor effect on the water evaporation rate and, consequently, on the time-dependent droplet radius. As a result, the term $\Delta D_{w}^{\mathrm{sol}}\left(r,t\right)$ in Eq. (23) can be safely neglected in the calculation of the droplet evaporation time and, thus, one can replace $D_{w}^{l}$ in the numerical calculations described in the previous section (and in supplementary material Sec. B) by $D_{w}^{\mathrm{sol},i}$ to estimate the droplet evaporation time in the presence of solutes and in the absence of the possibility of phase separation and crust formation. Also, Eq. (20), which neglects the concentration-dependence of the water diffusion, can be safely used to estimate the droplet evaporation time in case when $\beta c_{s}^{i}$ is small enough to not significantly change the initial water diffusion coefficient from its value in pure water, i.e., for $0.5\leq \frac{D_{w}^{\mathrm{sol},i}}{D_{w}^{l}}\leq 10$. For droplets that initially contain a relatively large solute fraction, especially solutes that dramatically decrease the water diffusion coefficient, such as saccharides,^81^ the significantly reduced initial water diffusivity inside the droplet is expected to slow down the evaporation process [see Fig. 5(b)] and, thus, Eq. (20) underestimates the droplet evaporation time.

So far, we examined various effects of nonvolatile solutes on the droplet evaporation process, without considering the possibility of crust formation (which is discussed next). Numerical examples for evaporation times obtained from the relevant equations are given in Table I for a droplet consisting of a NaCl solution with initial radius $R_{0}=50\;\mu m$ at 25 °C. According to this table, the presence of solutes on the one hand decreases the droplet evaporation time by producing a lower limit for the droplet size that can be reached by evaporation and by reducing evaporation cooling of the droplet. On the other hand, the evaporation time is found to increase due to the solute-induced water–vapor pressure reduction and due to the presence of local concentration gradients inside the droplet that arise from the finite diffusivity of solutes and water in the liquid phase. Overall, the latter two effects are dominant and thus the droplet evaporation time increases in the presence of solutes. Neglecting solute effects, therefore, causes an underestimate of the droplet evaporation time. Table I also shows that solute effects are more pronounced at high initial solute volume fractions.

**TABLE I.** The evaporation time $\tau_{\mathrm{ev}}$ calculated for a NaCl solution droplet with $R_{0}=50\;\mu m$ at 25 °C considering separately different solute effects.^a^

Combination of solute effects considered	$\Phi_{0}=0.01$ RH=0	$\Phi_{0}=0.01$ RH=0.5	$\Phi_{0}=0.01$ RH=0.75	$\Phi_{0}=0.1$ RH=0.5	$\Phi_{0}=0.1$ RH=0.75
— [Eq. (8)]	5.95 s	11.9 s	23.8 s	11.9 s	23.8 s
I [Eq. (7)]	5.7 s	11 s	21 s	7.8 s	10.9 s
I, II [Eq. (10)]	6.5 s	13.5 s	28.8 s	17.9 s	39 s
I, II, III [Eq. (3)]	6 s	13 s	28.6 s	15.35 s	34.4 s
I, II, III, IV [Eq. (20)]	6.7 s	14.2 s	30.5 s	18.6 s	41.1 s
I, II, III, IV, V [Eq. (C16)]	6.7 s	-	-	18.6 s	41.1 s

^a^ Each row corresponds to model calculations that address a combination of different solute effects on the droplet evaporation time: (I) the solute-induced droplet equilibrium size (II) the water vapor-pressure reduction in the presence of solutes (III) the solute-concentration dependence of evaporation cooling (IV) the evaporation-induced water concentration gradient inside the droplet, and (V) the solute-concentration dependence of the internal water diffusivity.

#### 2. Effect of phase separation and crust formation

The results presented so far are obtained without accounting for the possibility of crust formation. Depending on the evaporation rate, temperature, and type of the solutes, a solid solute crust can form, which dramatically affects the drying process and the time it takes for the droplet to reach the evaporation equilibrium state. It is, therefore, of importance to determine when a crust forms on the droplet surface and how it affects the droplet evaporation kinetics. It is also of interest to examine whether the concentration-dependent variation of the internal water diffusivity delays or accelerates the crust formation.

A solid crust is known to form as a consequence of solute crystallization which, depending on the nucleation mechanism,^82^ starts at a certain level of supersaturation. Here, we assume that a crust forms instantaneously when the solute concentration at the droplet surface $c_{s}(R)$ reaches the corresponding supersaturation concentration $c_{s}^{\text{max}}$. The crust can form only if $c_{s}^{\text{max}}$ is lower than the equilibrium solute concentration $c_{s}^{ev}=\Phi_{ev}/N_{A}v_{s}$, with $N_{A}$ being the Avogadro constant and $\Phi_{ev}=1-RH/\gamma$ being the equilibrium solute volume fraction. The threshold relative humidity below which crust formation can occur during the drying process is, therefore, given by

$${RH}^{*}=\gamma \left(1-c_{s}^{\text{max}}v_{s}N_{A}\right),$$ (24)

where $c_{s}^{\text{max}}v_{s}N_{A}$ expresses the maximal possible volume fraction of solutes before crust formation. For droplets consisting of a NaCl solution, crust formation is reported to occur once a supersaturation factor of 2.04 is achieved at the droplet surface, which corresponds to a supersaturation concentration of around $c_{s}^{\text{max}}=11.1\;M.$^8^ Crust formation is, therefore, a very probable phenomenon for droplets containing NaCl solution that occurs when the relative humidity is lower than ${RH}^{*}\approx 0.8$.

Another assumption made here is that the outer radius of the droplet remains constant once the crust has formed, meaning that the possibility of crust collapse [Fig. 1, scenario (e)] or wrinkling [Fig. 1, scenario (f)] is ignored. Accordingly, one can define the droplet equilibrium radius $R_{ev}$ as the larger of the radii at which the droplet reaches the evaporation-equilibrium state (in the absence of crust) and at which a crust first forms on the droplet surface. Neglecting the concentration dependence of the water diffusivity within the droplet, the equilibrium radius $R_{ev}$ follows from Eq. (B23) as

$$R_{ev}=R_{0}{\left(1+\frac{\left({\left(\frac{R_{i}^{*}}{R_{ev}}\right)}^{3}-3\left(\frac{R_{i}^{*}}{R_{ev}}\right)+2\right)\left(1-\Phi_{0}-\frac{RH}{\gamma }\right)}{2\Phi_{0}\left(\left(\frac{\alpha }{\gamma }-1+\alpha \varepsilon_{T}\varepsilon_{c}\left(1-\Phi_{0}\right)\right)\left(\frac{R_{i}^{*}}{R_{ev}}\right)+1\right)}\right)}^{-\frac{1}{3}},$$ (25)

where $R_{i}^{*}$ is the radius of the internal core at the moment when the droplet reaches its final equilibrium size $R_{ev}$. According to the water concentration profile [Eq. (14)], the ratio $R_{i}^{*}/R_{ev}$ is given by

$$\frac{R_{i}^{*}}{R_{ev}}={\left(1-\frac{\frac{\alpha }{\gamma }\left(\Phi_{0}-\Phi_{\text{max}}\right)\left(1+\gamma \varepsilon_{T}\varepsilon_{c}\left(1-\Phi_{0}\right)\right)}{1-\Phi_{\text{max}}-\frac{RH}{\gamma }}\right)}^{-1},$$ (26)

with $\Phi_{\text{max}}$ being the maximal possible volume fraction of solutes, i.e., the smaller of $c_{s}^{\text{max}}v_{s}N_{A}$ and $\Phi_{ev}=1-RH/\gamma$. In the case when the drying process ends before a crust forms (i.e., when $1-RH/\gamma <c_{s}^{\text{max}}v_{s}N_{A}$), the above equations yield $R_{i}^{*}=0$ and, consequently, $R_{ev}=R_{0}{\left(\frac{\Phi_{0}}{1-RH/\gamma }\right)}^{1/3}$, which is equal to what we previously obtained in the absence of crust formation [see Eq. (4)].

**FIG. 7.**
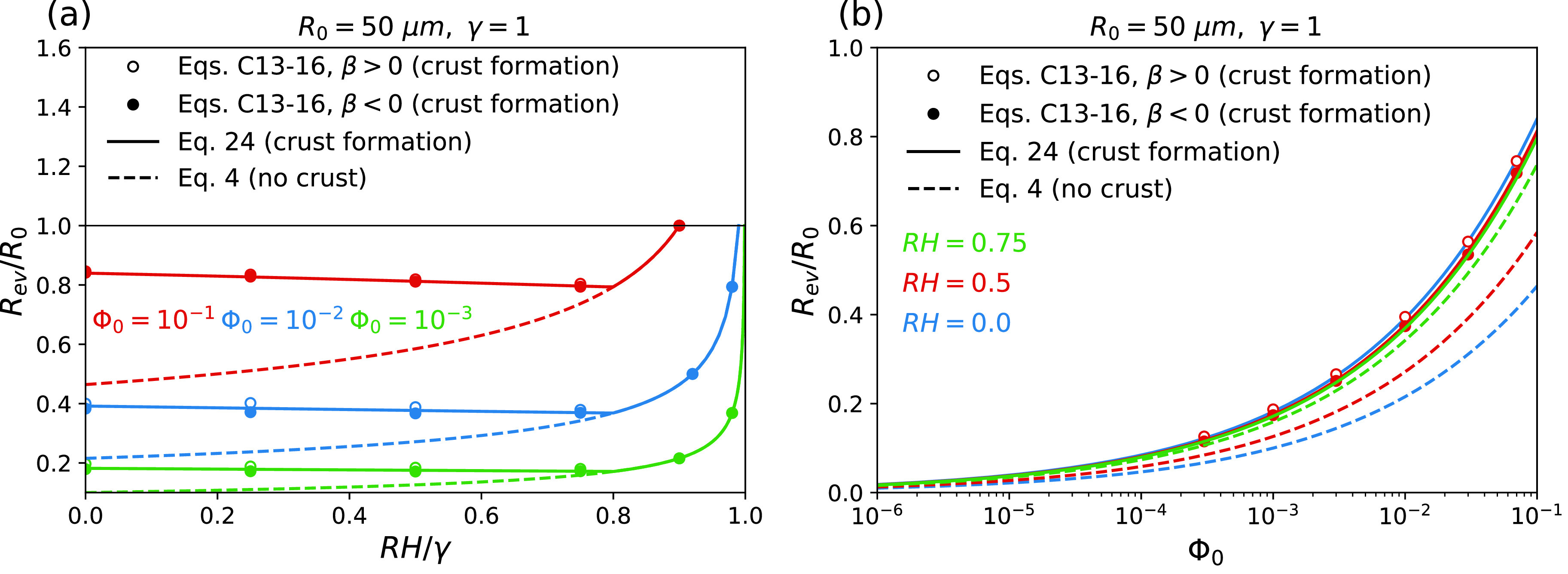
Variation of the droplet equilibrium radius $R_{ev}$ as a function of (a) the relative humidity RH divided by the water activity coefficient γ and (b) the initial volume fraction of solutes $\Phi_{0}$. Broken lines show the results from Eq. (4) that neglects the possibility of crust formation and solid lines show the results from Eq. (25), which is derived assuming that a solid crust forms on the droplet surface when the solute concentration at the surface reaches a critical value, taken here as $c_{s}^{\text{max}}=11.1\;M$, which is the critical supersaturation concentration at which crystallization occurs at the surface of an evaporating droplet containing a NaCl solution.^8^ The results obtained from the numerical method described in supplementary material Sec. C.2, which accounts for the solute-concentration dependence of the internal water-diffusion coefficient, are shown by circles. These results are calculated using $\beta =\pm 0.065\;M^{-1}$, $v_{s}=v_{w}=3\times 10^{-29}\;m^{3}$ [see Eq. (22)], and $D_{w}^{l}=D_{w}^{l,25{}^{\circ}C}=2.3\times 10^{-9}\;m^{2}/s$ (see the Nomenclature).

According to Fig. 7(a), when RH/γ is lower than the threshold value given by Eq. (24), a crust forms at the surface of the droplet and thus the droplet equilibrium size calculated from Eq. (25) is greater than what would be expected in the absence of a crust. In this case, an increase in RH/γ slightly decreases $R_{ev}$, which can be attributed to the reduced water evaporation rate in a humid environment or in solutions with low water-activity coefficients. Indeed, when the evaporation rate decreases, the solute particles at the droplet surface have more time for diffusional motion toward the center of the droplet and thereby, the surface concentration rises more slowly. This in turn causes a slight delay in the crust formation and leads to a smaller droplet equilibrium size. When RH/γ exceeds the threshold amount given by Eq. (24), however, the evaporation rate becomes so small that a crust no longer forms at the droplet surface. In this case, the droplet equilibrium radius rapidly increases with RH/γ, as expected in the absence of crust formation [see Eq. (4)]. Figure 7(b) indicates that an increase in the initial solute volume fraction increases the droplet equilibrium size, as expected.

To examine how the solute-concentration dependence of the internal water diffusivity affects the results, the equilibrium radius is calculated using the numerical method described in the supplementary material Sec. C.2 for both β>0 and β<0, i.e., considering that $D_{w}^{l,\mathrm{sol}}$ decreases or increases with the solute concentration [see Eq. (22)]. The results are shown by open and solid symbols in Fig. 7 and demonstrate that the solute-concentration dependence of the internal water diffusivity can slightly accelerate the crust formation when β>0, or delay it when β<0. Neglecting such dependence, however, does not make a significant error in the results.

The next step would be to model the evaporation process in the presence of a crust. Due to the diversity of possible crust structures and lack of experimental data on the crust properties, this step is difficult. As discussed in Sec. 1, depending on the type of solutes, the crust formed during the drying process might be a dry crust, which forms due to crystallization of salts in a salt solution, or a gel-like wet skin, which is expected to form on the surface of polymer-containing droplets. In the case of dry salt crusts, the evaporation process would continue through capillary action.^70,76^ To model the evaporation in the presence of such a crust, one would need to know the crust porosity, its characteristic pore size, and the amount of water vapor or liquid water located inside the pores. In the case of wet crusts, the evaporation continues through water diffusion in the gel phase, which is easier to model. However, one would still need to know the variation of the water activity coefficient with the local concentration and the water diffusion constant within the crust. In addition, to provide a realistic model for water evaporation after the crust has formed, one would need to consider the other possible fates for the crust, such as crust collapse [Fig. 1, scenario (e)] and wrinkling [Fig. 1, scenario (f)]. Therefore, modeling of the water evaporation process after crust formation is left for future research.

## III. CONCLUSION

This paper reports analytical and semi-analytical solutions of the coupled heat and mass diffusion equations and investigates the effects of nonvolatile solutes on water evaporation from solute-containing aqueous droplets. The model accounts for evaporation cooling, internal water concentration gradients, solute-induced water vapor-pressure reduction, and the solute-concentration dependence of the water diffusivity inside the droplet. For this, the evaporating droplet is assumed to contain two regions: an outer shell, where the liquid phase exhibits an inhomogeneous water concentration, and an inner core, where the water concentration profile is uniform. Accordingly, the water evaporation is considered as a two-stage process. In the first stage, the outer shell grows from the surface toward the core of the droplet. When the shell covers almost the entire droplet, evaporation turns into the second stage, where the water concentration in the inner core gradually decreases to its equilibrium value. Our model accounts for solute-induced deviations from the classical *R*^2^-law, which assumes that the droplet temperature and thereby also the evaporation rate remains constant during the drying process.

Our investigations indicate that the presence of nonvolatile solutes within a drying droplet leads to opposing effects on the evaporation speed that partly compensate each other. The dominant effect is the water vapor-pressure reduction due to the reduced water concentration in the presence of solutes, which considerably slows down the evaporation process. Finite diffusivity of water and solute molecules within the droplet, which gives rise to a water concentration gradient in the droplet, further intensifies the solute-induced slowing down of the droplet evaporation. On the other hand, the presence of solutes produces a lower limit for the droplet radius that can be reached by evaporation, which tends to reduce the droplet evaporation time. Furthermore, the evaporation-induced increase in the solute concentration inside the droplet reduces the evaporation cooling, which slightly accelerates the droplet evaporation process. According to our results, the latter two effects are subdominant compared to the first two, but still considerable, meaning that the droplet evaporation time effectively increases in the presence of nonvolatile solutes.

The solute-induced slowing down of the droplet evaporation process can be intensified by any factor that decreases the water diffusivity inside the droplet, such as the presence of strongly hydrated solutes. The inhomogeneous diffusivity profile inside the droplet arising from the solute-concentration dependence of the water diffusion coefficient influences the droplet evaporation process. Our results, however, reveal that this factor plays only a minor role in determining the droplet evaporation rate and can be safely neglected. In fact, the presence of strongly hydrated solutes on the one hand decreases the water-diffusion coefficient at the droplet surface due to the increased solute concentration at the surface. On the other hand, the presence of such solutes decreases the growth rate of the outer shell, where the water concentration is inhomogeneous, which leads to an increase in the water concentration gradient at the droplet surface. As a result, the water evaporation flux, which is the product of the water-diffusion coefficient and the water concentration gradient at the droplet surface, remains almost independent of the solute-induced variations of the internal water diffusivity.

The physical–chemical effects that control the droplet evaporation process are dominated by various parameters, among which the initial solute volume fraction and the ambient relative humidity play key roles in determining the droplet evaporation time and the final equilibrium size of the evaporating droplet. The droplet evaporation time increases in humid environments due to the reduced evaporation rate. The effect of the initial solute volume fraction $\Phi_{0}$ is more complicated. The droplet evaporation time follows a non-monotonic profile as a function of $\Phi_{0}$ and exhibits a maximum at a certain value of $\Phi_{0}$ that is around half of the solute volume fraction in the equilibrium state. This non-monotonic behavior is found to arise from logarithmic osmotic contributions in the droplet evaporation time. In the absence of crust formation, the droplet equilibrium size increases when either the initial solute volume fraction or the relative humidity increases.

When the solute concentration at the droplet surface exceeds a critical supersaturation concentration, a solid crust forms on the droplet surface, which decisively influences the evaporation kinetics. The crust formation also affects the morphology of the dry residue that remains after evaporation by producing a hollow particle with a size larger than expected in the absence of crust formation. Assuming that the droplet retains its spherical shape during the evaporation process and neglecting the possibility of crust collapse or wrinkling, the outer radius of the hollow particle formed after crust formation is predicted analytically. Our results reveal three factors that can delay the crust formation and thus reduce the final size of the hollow particle: a decrease in the initial solute volume fraction, an increase in the relative humidity, or a decrease in the water activity coefficient. The latter factor represents a reduced volatility of water molecules due to non-ideal effects caused by water–solute and solute–solute interactions. Our results also indicate that the crust formation is slightly accelerated in the presence of solutes that decrease the internal water diffusivity, e.g., strongly hydrated solutes, and slightly delayed in the presence of solutes that increase the internal water diffusivity, e.g., weakly hydrated solutes. This effect is, however, insignificant and can be safely neglected.

Although the present study provides insight into the effects of nonvolatile solutes on the evaporation process of solute-containing droplets, further work is required to answer a number of important open questions: (I) How does the water activity coefficient vary during the lifetime of the droplet and how do non-ideal effects due to solute–water interactions affect the drying process? (II) How does the evaporation process continue after crust formation? What is the exact mechanism of water evaporation in the presence of a dry crust? Does it involve capillary action? If so, more information about the crust porosity, its characteristic pore size, and the amount of water located inside the pores is required. (III) What happens after the formation of a gel-like skin that includes saturated polymers?

## SUPPLEMENTARY MATERIAL

See the supplementary material for the calculation of the droplet evaporation time with and without considering the evaporation cooling effect, the evaporation-induced concentration gradient inside the droplet, and the solute-concentration dependence of the internal water diffusivity.

## Data Availability

The data that support the findings of this study are openly available in Refubium at http://doi.org/10.17169/refubium-30739, Ref. 83.
